# Polyspecific Intravenous Immunoglobulin in Clindamycin-treated Patients With Streptococcal Toxic Shock Syndrome: A Systematic Review and Meta-analysis

**DOI:** 10.1093/cid/ciy401

**Published:** 2018-05-18

**Authors:** Tom Parks, Clare Wilson, Nigel Curtis, Anna Norrby-Teglund, Shiranee Sriskandan

**Affiliations:** 1 London School of Hygiene and Tropical Medicine, United Kingdom; 2 Imperial College London, United Kingdom; 3 Royal Children’s Hospital, Melbourne, Victoria, Australia; 4 Karolinska Institutet, Stockholm, Sweden

**Keywords:** Group A streptococcus, *Streptococcus pyogenes*, intravenous immunoglobulin, streptococcal toxic shock syndrome, necrotising fasciitis

## Abstract

We evaluated the effect of intravenous immunoglobulin (IVIG) on mortality in clindamycin-treated streptococcal toxic shock syndrome using a meta-analysis. In association with IVIG, mortality fell from 33.7% to 15.7% with remarkable consistency across the single randomized and four nonrandomized studies.

Streptococcal toxic shock syndrome (STSS) is a complication of invasive group A streptococcal (IGAS) infection, characterized by hypotension and end-organ failure, often with immunological manifestations such as rash [1]. Notwithstanding the formal case definition, it should be noted that shock in a patient with IGAS infection will almost always represent STSS [2]. Complicating IGAS infection in approximately 10% of cases, STSS is thought to be triggered in part by superantigens and other bacterial virulence factors. The STSS-associated mortality rate is substantial, exceeding 25% within the first 24 hours in some studies [3]. In addition, STSS is associated with substantial morbidity, with most cases requiring intensive care.

Polyspecific intravenous immunoglobulin (IVIG) is recommended by some experts as an adjunctive treatment for STSS, not least because of laboratory data indicating potentially beneficial effects, including neutralization of superantigens and enhanced bacterial clearance [4]. However, the use of IVIG for STSS has been difficult to evaluate clinically; the only randomized controlled trial (RCT) was stopped early owing to slow recruitment [5]. Although a small number of nonrandomized studies have been reported, the interpretation of these data is complicated by the inherent risk of bias, the variable inclusion criteria, and the inconsistent use of clindamycin, which is widely advocated as an adjunct to penicillin. We undertook a systematic review of randomized and nonrandomized studies that evaluated the use of adjunctive IVIG in STSS. We then performed a meta-analysis of the effect of IVIG on mortality rates in the subgroup of patients with STSS whose antibiotic therapy included clindamycin.

## METHODS

We searched English-language entries in MEDLINE and EMBASE since 1980, using the terms “streptococcus” OR “streptococcal” AND “intravenous immunoglobulin” OR “IVIG” (Supplementary Figure 1). We also searched reference lists of short-listed articles. We included studies that evaluated the relationship between IVIG and mortality in patients with STSS prospectively identified using the consensus criteria [2]. We excluded studies that were retrospective and did not detail the use of clindamycin or did not define STSS. Eligibility assessment and data extraction were done without blinding by 2 of the authors (T. P. and C. W.). We also assessed the risk of bias, using tools published by the Cochrane Collaboration. In addition, we contacted the authors of eligible studies, including unpublished abstracts, to request a breakdown of all results by use of clindamycin. Our primary measure of treatment effect was the risk ratio (RR) for death at 30 days, calculated with its standard error for the subgroup of patients with STSS who received clindamycin. We then performed a meta-analysis using a random-effects model and assessed heterogeneity using the *I*^2^ statistic. All analyses were done using Stata software (version 12.1; StataCorp).

## RESULTS

The search, which was last updated on 31 December 2017, revealed 412 articles after removal of duplicates (Supplementary Figure 2). Of 14 short-listed articles, 1 randomized [5] and 4 nonrandomized studies [6–9] met the inclusion criteria (Supplementary Table 1 and Supplementary Table 2). The included studies were undertaken between 1992 and 2009 in Northern Europe, Canada, and Australia. The randomized study compared IVIG with placebo and the nonrandomized studies compared IVIG with standard care. One of the nonrandomized studies used historical controls [6] and the other 3 used concurrent patients who did not receive IVIG as controls [7–9]. Across all 5 studies, IVIG was administered to 70 and not administered to 95 of the patients with STSS treated with clindamycin (Supplementary Table 3). The overall mortality rate was 26.1%, ranging between 14.3% and 40.5% in the individual studies.

We found risk of bias across several domains in the nonrandomized studies (Supplementary Table 4). In particular, we noted at least moderate risk of bias due to baseline differences between IVIG-treated case patients and controls. Although adjusted analyses were reported, it is likely that some baseline confounding persisted, not least because the small sample sizes limited the utility of multivariate regression. Despite limiting our analyses to the subgroups treated with clindamycin, we expect that some of this bias remained in our analyses. In addition, 2 of these studies collected some information retrospectively, using questionnaires, with the potential for selection bias. Furthermore, 3 of the studies provided no details of IVIG dosing, potentially introducing classification bias. Separately, a funnel plot of the 4 nonrandomized studies—using all reported data rather than the subset analyzed here—suggests the possibility of reporting bias, although it is difficult to interpret the plot with so few studies (Supplementary Figure 3). In contrast, we found limited risk of bias in the randomized study (Supplementary Table 5).

**Figure 1.**
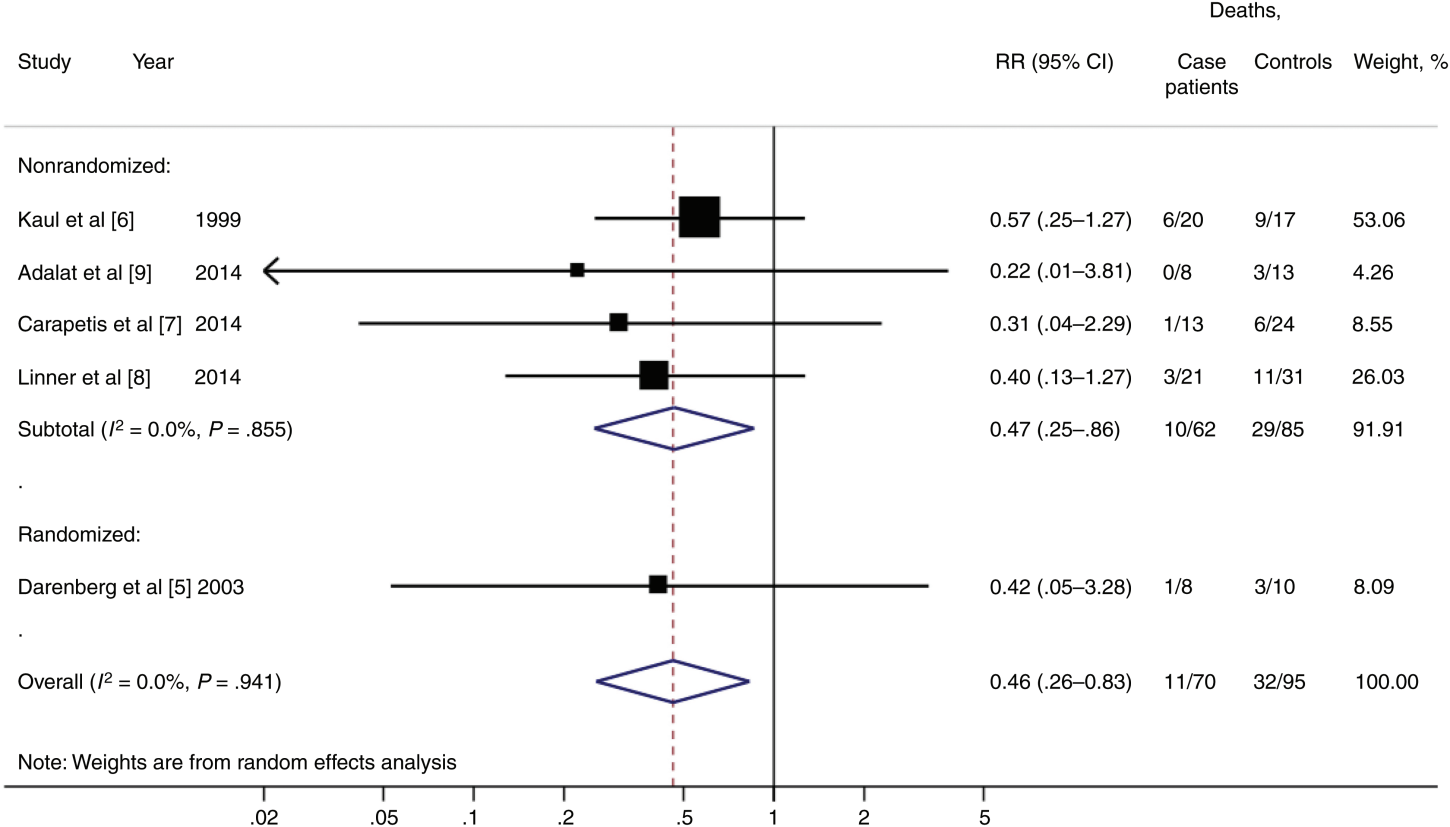
Forest plot showing the estimated risk ratio for mortality with or without intravenous immunoglobulin in clindamycin-treated streptococcal toxic shock syndrome.

Abbreviations: CI, confidence interval; RR, risk ratio.

In all 5 studies, administration of IVIG in the clindamycin-treated subgroup was associated with lower mortality rates, but it did not reach statistical significance in isolation in any of the studies (Figure 1; Supplementary Table 6). However, in the pooled analysis, administration of IVIG was associated with a reduction in mortality rate from 33.7% to 15.7% (RR, 0.46; 95% confidence interval, .26–.83; *P* = .01), with negligible heterogeneity (*I*^2^ = 0%). The pooled result of the nonrandomized studies (RR, 0.47; 95% CI, .25–.86) was remarkably consistent with the effect size estimate of the RCT (RR, 0.42; 95% CI, .05–3.28).

## DISCUSSION

This systematic review and meta-analysis provide evidence that administration of adjunctive IVIG to clindamycin-treated patients with STSS is associated with a statistically significant reduction in mortality rate. Crucially, our analysis disentangles the effects of clindamycin from those of IVIG, which has sometimes been problematic [6, 7]. Our results therefore corroborate the findings of the study by Linnér et al [8], the largest of the 3 more recent nonrandomized studies, which suggested that both clindamycin and IVIG were beneficial. Moreover, by limiting the analysis to clindamycin-treated subgroups, we provide a more informative effect size estimate than those derived from the individual datasets. Overall, our results imply that up to 1 additional death could be prevented for every 6 clindamycin-treated patients with STSS given IVIG.

Three of the studies we excluded are worthy of further discussion, not least because their main results seem to contradict our findings. The first prospectively assessed the efficacy of IVIG in patients with IGAS infection, with or without STSS, who were admitted to the intensive care units at 4 tertiary hospitals in Canada [10]. Unfortunately, the authors of that report were unable to provide us with the results for the subset of patients with STSS treated with clindamycin. Thus, although IVIG had no effect on IGAS-associated mortality rates overall, its impact in the subset of patients with STSS remains unknown.

The second study retrospectively identified patients with STSS admitted to tertiary pediatric hospitals in the United States using discharge diagnoses coded according to the *International Classification of Diseases, Ninth Revision* [11]. Accordingly, this study is highly likely to have included patients with diagnoses other than STSS, a group that would have been both less likely to receive IVIG and less likely to die than those with STSS. The third study respectively identified patients with necrotizing fasciitis and vasopressor-dependent shock from 121 hospitals in the Untied States [12]. In a propensity-matched analysis based on 322 patients, those authors found that IVIG had no effect on mortality. However, addition to our meta-analysis of data from 49 patients with coding for *S. pyogenes* and clindamycin (Supplementary Table 7) had a negligible effect on our results (Supplementary Figure 4).

Our study has 3 main limitations. First, despite pooling 5 studies, our analysis remains small, and consequently our effect size estimate lacks precision. Second, despite limiting the meta-analysis to the clindamycin-treated subgroup, there is a sizeable risk that the baseline characteristics differed between patients given and those not given IVIG. For example, in the study by Linnér et al [8], there were differences at baseline in terms of age, comorbid conditions, and presence of necrotizing fasciitis, all of which were associated with increased risk of death. Nonetheless, we predict that IVIG would be administered more frequently to the most unwell patients, thereby introducing any bias toward a null effect. That said, although the similarity of the effect size estimate in the single RCT and 4 nonrandomized studies is reassuring, it remains plausible that the reduction in mortality rates associated with IVIG in this analysis is due to confounding.

Third, relatively limited information was available regarding the use of antibiotics other than clindamycin. This issue could theoretically bias our results in favor of IVIG if certain potentially beneficial antibiotics, including penicillin, were used more often with IVIG. It is noteworthy, however, that the antibiotic regimen in the RCT was prespecified [5] and all but 1 patient in the study by Linnér et al received a β-lactam agent [8]. Fourth, we were unable to address a number of outstanding questions, including the optimum dosing and timing of IVIG. Ultimately, therefore, in the absence of sufficiently sized RCTs, a meta-analysis of observational studies may be the best means available to evaluate such an intervention. Looking forward, establishment of an international registry of STSS cases may provide more robust data to inform management of this devastating condition.

In conclusion, our study helps address doubt surrounding the use of IVIG in STSS. It also highlights the utility of synthesizing findings from small nonrandomized studies in the absence of large-scale trials. Overall, given the high morbidity and mortality rates associated with STSS, we support the use of IVIG as an adjunctive treatment for STSS, a recommendation that applies to the vast majority of patients with IGAS infection complicated by shock.

## Supplementary Data

Supplementary materials are available at *Clinical Infectious Diseases* online. Consisting of data provided by the authors to benefit the reader, the posted materials are not copyedited and are the sole responsibility of the authors, so questions or comments should be addressed to the corresponding author.

Supplementary Material

## Notes

***Acknowledgments.*** We thank the authors of the underlying studies who responded to our requests for a breakdown of their results by use of clindamycin.

***Disclaimer.*** The views expressed are those of the authors and not necessarily those of the National Health Service, the National Institute for Health Research, the Department of Health, or Public Health England.

***Financial support.*** This work was supported by the Medical Research Council (grant G1100449 to T. P.), the National Institute for Health Research (grant ACF-2016-20-001 to T. P.), the National Institute for Health Research Health Protection Research Unit in Antimicrobial Resistance and Healthcare Associated Infection in partnership with Public Health England (grant HPRU-2012–10047), and the National Institute for Health Research Imperial Biomedical Research Centre.

***Potential conflicts of interest.*** All authors: No reported conflicts. All authors have submitted the ICMJE Form for Disclosure of Potential Conflicts of Interest. Conflicts that the editors consider relevant to the content of the manuscript have been disclosed.

## References

[1] Stevens DL Streptococcal toxic-shock syndrome: spectrum of disease, pathogenesis, and new concepts in treatment. Emerg Infect Dis 1995; 1:69–78. 8903167 10.3201/eid0103.950301 PMC2626872

[2] The Working Group on Severe Streptococcal Infections. Defining the group A streptococcal toxic shock syndrome. Rationale and consensus definition. JAMA 1993; 269:390–1. 8418347

[3] Lamagni TL, Neal S, Keshishian C, et al Predictors of death after severe *Streptococcus pyogenes* infection. Emerg Infect Dis 2009; 15:1304–7. 19751599 10.3201/eid1508.090264

[4] Sriskandan S, Ferguson M, Elliot V, Faulkner L, Cohen J Human intravenous immunoglobulin for experimental streptococcal toxic shock: bacterial clearance and modulation of inflammation. J Antimicrob Chemother 2006; 58:117–24. 16670109 10.1093/jac/dkl173

[5] Darenberg J, Ihendyane N, Sjölin J, et al; StreptIg Study Group Intravenous immunoglobulin G therapy in streptococcal toxic shock syndrome: a European randomized, double-blind, placebo-controlled trial. Clin Infect Dis 2003; 37:333–40. 12884156 10.1086/376630

[6] Kaul R, McGeer A, Norrby-Teglund A, et al; Canadian Streptococcal Study Group.Intravenous immunoglobulin therapy for streptococcal toxic shock syndrome–a comparative observational study.. Clin Infect Dis 1999; 28:800–7. 10.1086/515199 10825042

[7] Carapetis JR, Jacoby P, Carville K, Ang SJ, Curtis N, Andrews R Effectiveness of clindamycin and intravenous immunoglobulin, and risk of disease in contacts, in invasive group A streptococcal infections. Clin Infect Dis 2014; 59:358–65. 24785239 10.1093/cid/ciu304

[8] Linnér A, Darenberg J, Sjölin J, Henriques-Normark B, Norrby-Teglund A Clinical efficacy of polyspecific intravenous immunoglobulin therapy in patients with streptococcal toxic shock syndrome: a comparative observational study. Clin Infect Dis 2014; 59:851–7. 24928291 10.1093/cid/ciu449

[9] Adalat S, Dawson T, Hackett SJ, Clark JE; British Paediatric Surveillance Unit Toxic shock syndrome surveillance in UK children. Arch Dis Child 2014; 99:1078–82. 24790135 10.1136/archdischild-2013-304741

[10] Mehta S, McGeer A, Low DE, et al Morbidity and mortality of patients with invasive group A streptococcal infections admitted to the ICU. Chest 2006; 130:1679–86. 17166982 10.1378/chest.130.6.1679

[11] Shah SS, Hall M, Srivastava R, Subramony A, Levin JE Intravenous immunoglobulin in children with streptococcal toxic shock syndrome. Clin Infect Dis 2009; 49:1369–76. 19788359 10.1086/606048 PMC2761980

[12] Kadri SS, Swihart BJ, Bonne SL, et al Impact of intravenous immunoglobulin on survival in necrotizing fasciitis with vasopressor-dependent shock: a propensity-score matched analysis from 130 US hospitals. Clin Infect Dis 2016; 64:877–85. 10.1093/cid/ciw871 PMC5850528 28034881

