# Supplementary material for: Polyspecific Intravenous Immunoglobulin in Clindamycin-treated Patients With Streptococcal Toxic Shock Syndrome: A Systematic Review and Meta-analysis
Source: Clin Infect Dis. 2018 May 18;67(9):1434–6. doi: 10.1093/cid/ciy401 (PMC6186853; doi:10.1093/cid/ciy401)
Supplement: Supplementary Material [file ciy401_suppl_supplementary_material.pdf]

Search strategy: MEDLINE/EMBASE (OVID)
1. (streptococcus or streptococcal).af.
2. limit 1 to english language
3. limit 2 to yr=“1980 - 2017”
4. (intravenous immunoglobulin or ivig).af.
5. 3 and 4

Supplementary Figure 1. Search strategy

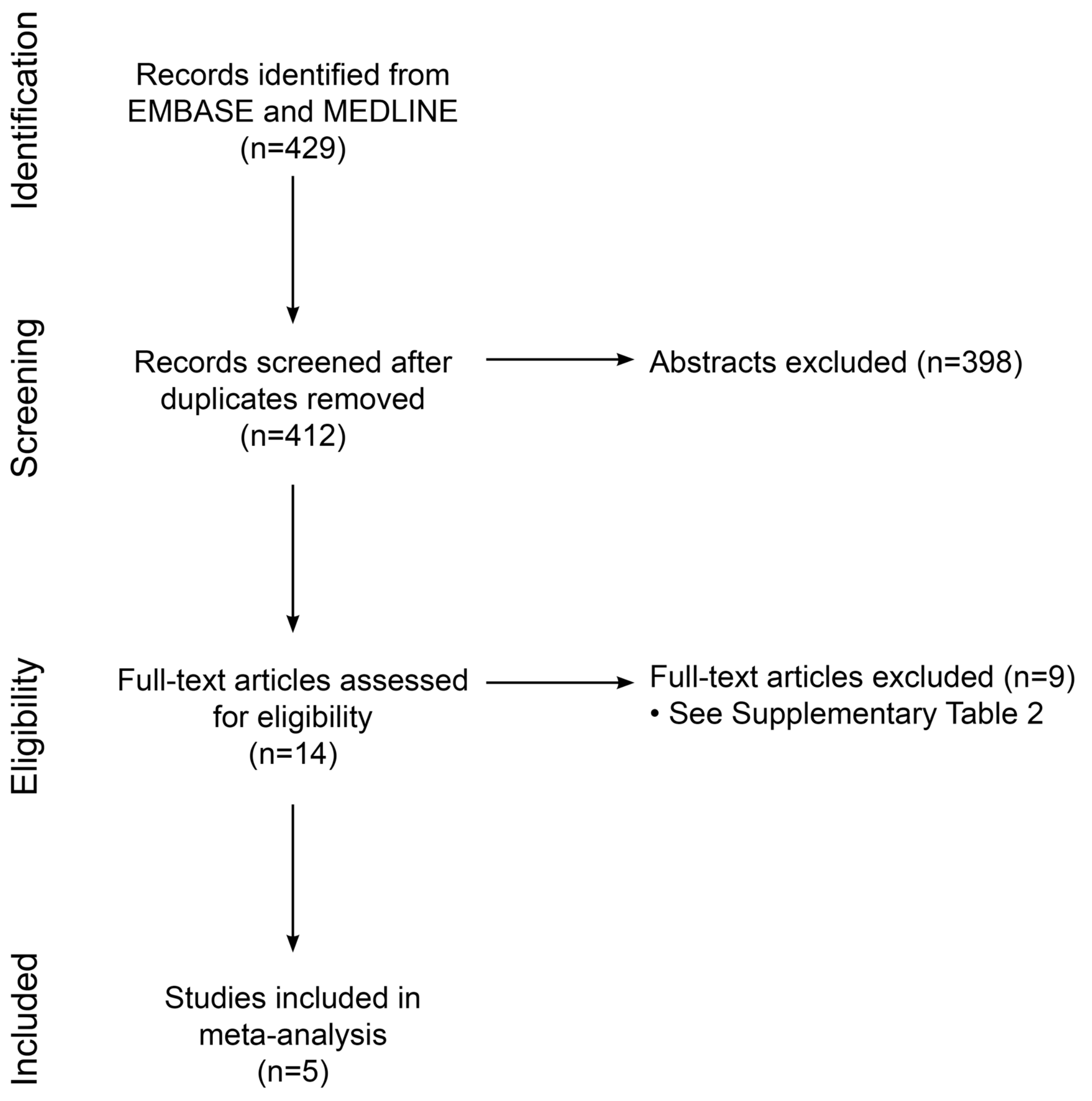

Supplementary Figure 2. Flow diagram showing study selection

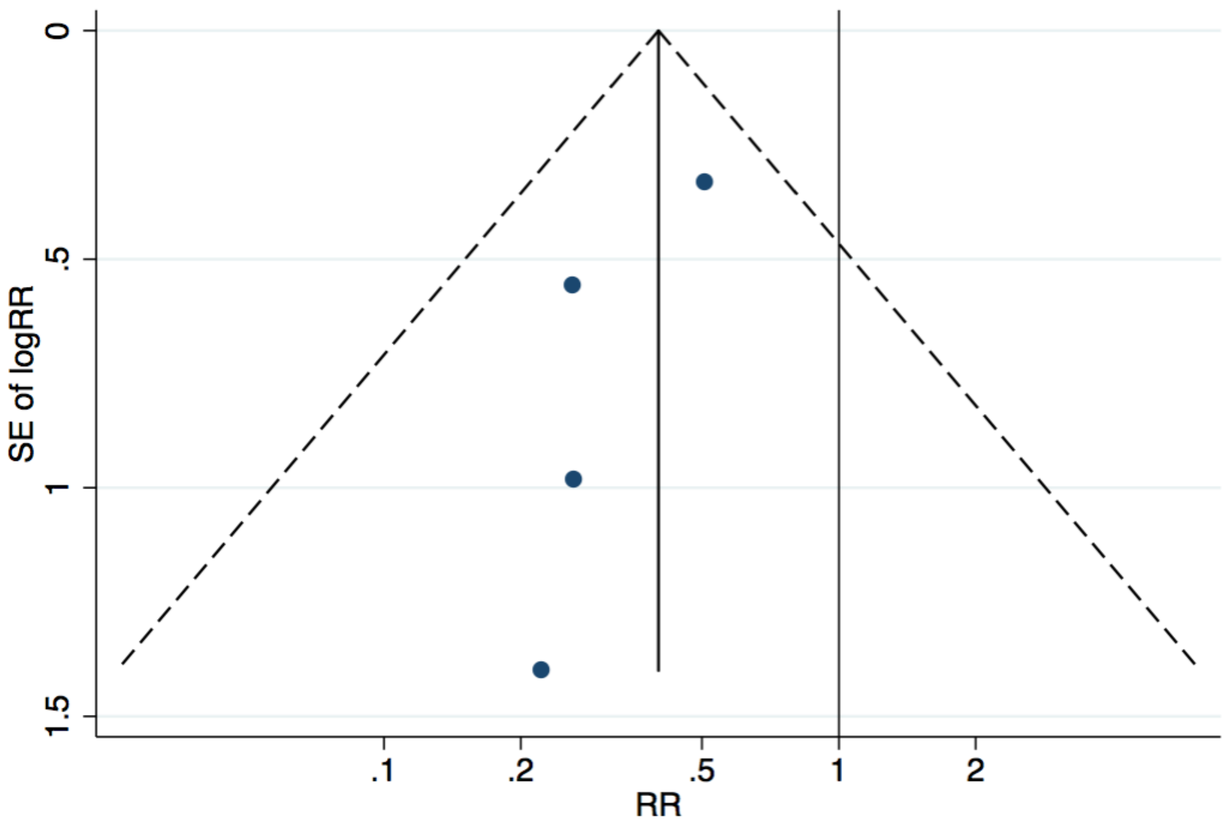

Supplementary Figure 3. Funnel plot for the non-randomised studies showing the unadjusted risk ratio for mortality with and without IVIG calculated from the full reported dataset (i.e. irrespective of clindamycin, GAS or STSS)

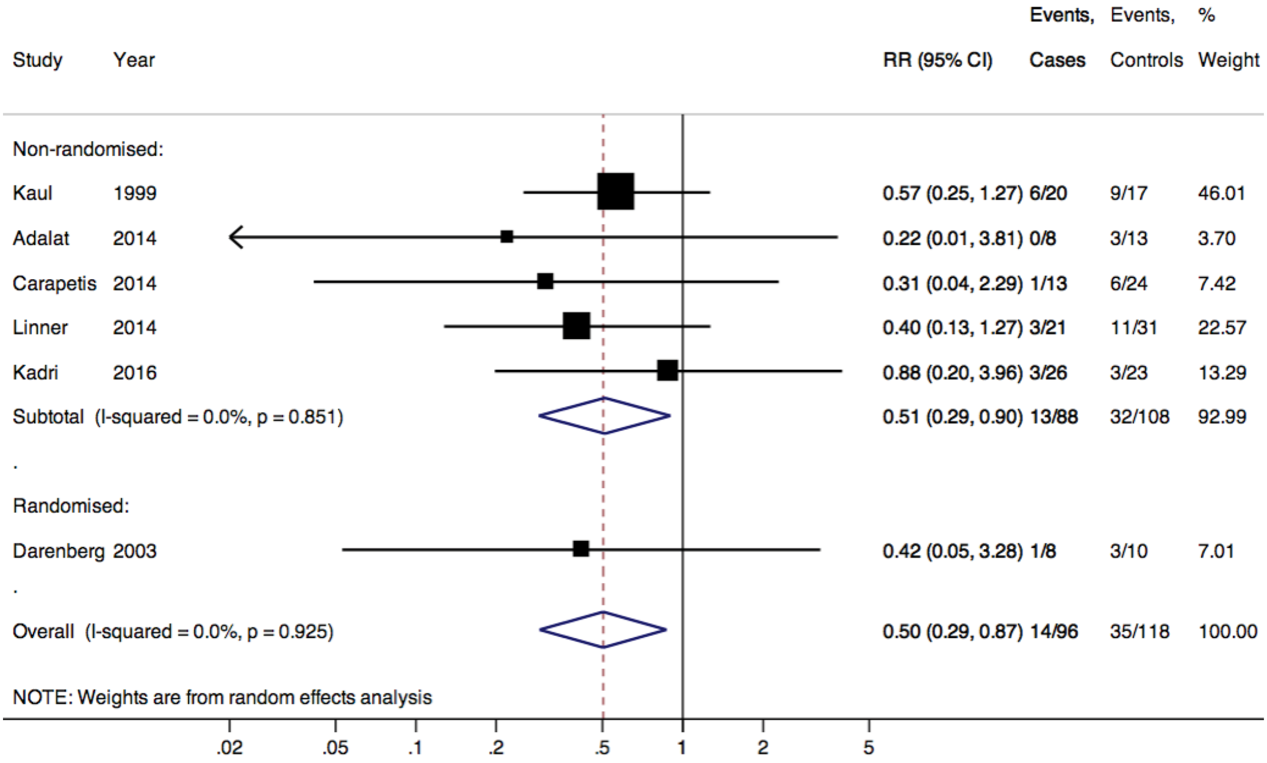

Supplementary Figure 4. Forest plot showing the estimated risk ratio for mortality with and without IVIG in clindamycin-treated STSS with the addition of data from the propensity-matched case-control analysis by Kadri *et al.* [12].

Supplementary Table 1. Summary of the included studies

Study	Location	Year	Design	Age range	Outcome	Intervention	Cases	Controls	Ref.
Kaul *et al.* 1999	Canada	1992-1995	Non-randomised with historical controls	Adults (mean 56.8 years; range not reported)	Mortality at 30 days	IVIG vs standard care	GAS-associated STSS patients identified prospectively through surveillance treated with IVIG	GAS-associated STSS patients identified prospectively through earlier surveillance not treated with IVIG	[6]
Darenberg *et al.* 2003	Sweden, Norway, Finland, Netherlands	1999-2001	Randomised double-blind, placebo-controlled trial	Adults (28-83 years)	Mortality at 28 days	IVIG vs placebo (equal vol. 1% albumin)	STSS patients enrolled on the basis of suspicion of GAS infection randomised to IVIG	STSS patients enrolled on the basis of suspicion of GAS infection randomised to placebo	[5]
Carapetis *et al.* 2014	Australia	2002-2004	Non-randomised with concurrent controls	Adults and children (3-88 years)	Mortality at 30 days	IVIG vs standard care	Severe IGAS patients identified prospectively through surveillance treated with IVIG	Severe IGAS patients identified prospectively through surveillance not treated with IVIG	[7]
Linnér *et al.* 2014	Sweden	2002-2004	Non-randomised with concurrent controls	Adults (31-92 years)	Mortality at 28 days	IVIG vs standard care	GAS-associated STSS patients identified prospectively through surveillance treated with IVIG	GAS-associated STSS patients identified prospectively through surveillance not treated with IVIG	[8]
Adalat *et al.* 2014	UK	2008-2009	Non-randomised with concurrent controls	Children (0-15 years)	Mortality at 28 days	IVIG vs standard care	GAS-associated STSS patients identified prospectively through surveillance treated with IVIG	GAS-associated STSS patients identified prospectively through surveillance not treated with IVIG	[9]

Supplementary Table 2. Summary of the excluded studies

Study	Location	Year	Principal Reason(s) for Exclusion	Total IGAS	Proportion STSS	Ref.
Haywood *et al.* 1999	Canada	1995-1997	Less than 10 STSS cases	20	5/20 (25%)	[13]
Huang *et al.* 2001	Taiwan	1995-2000	Retrospective study	76	12/76 (16%)	[14]
Norrby-Teglund *et al.* 2005	Canada	1996-2002	Less than 10 STSS cases	7	6/7 (86%)	[15]
Mehta *et al.* 2006	Canada	1992-2002	Subset data unavailable	62	34/62 (55%)	[10]
Aronoff & Mulla 2008	USA	1996-2001	Retrospective study<br>Less than 10 STSS cases	7	1/7 (14%)	[16]
Shah *et al.* 2009	USA	2003-2007	Retrospective study<br>Consensus criteria not used	192	192/192 (100%)	[11]
McViety *et al.* 2014	UK	2008-2013	Retrospective study<br>No deaths in clindamycin treated cases	23	17/23 (74%)	[17]
Chen *et al.* 2016	Australia	2003-2014	Retrospective study<br>No deaths in clindamycin treated cases	19	19/19 (100%)	[18]
Kadri *et al.* 2016*	USA	2010-2014	Retrospective study<br>Consensus criteria not used	228	228/228(100%)	[12]

*In total 50 of 228 (21.9%) patients with coding for GAS in the entire study were included in the propensity-matched analysis.

Supplementary Table 3. Derivation of subgroup of interest from included studies

Study	Total IGAS	Proportion STSS	Proportion treated with clindamycin	Proportion treated with clindamycin & IVIG (i.e. cases)	Proportion treated with clindamycin & not IVIG (i.e. controls)	Ref.
Kaul *et al.* 1999*	53	53/53 (100%)	37/53 (37%)	20/37 (54%)	17/37 (46%)	[6]
Darenberg *et al.* 2003	18	18/18 (100%)	18/18 (100%)	8/18 (44%)	10/18 (56%)	[5]
Carapetis *et al.* 2014†	84	49/84 (58%)	37/49 (76%)	13/37 (35%)	24/37 (65%)	[7]
Linnér *et al.* 2014	746	67/746 (9%)	52/67 (78%)	21/52 (40%)	31/52 (60%)	[8]
Adalat *et al.* 2014	29	29/29 (100%)	21/29 (72%)	8/21 (38%)	13/21 (62%)	[9]

*Data on clindamycin not available for one control who died.

†Includes severe IGAS patients with and without STSS; 49 classified as having STSS.

Supplementary Table 4. Risk of bias in the non-randomised studies [19]

Study	Confounding at baseline*	Bias in selection	Bias in classification	Bias in deviations	Bias in missingness	Bias in measurement	Bias in reporting	Ref.
Kaul *et al.* 1999	Moderate	Low	Low	Low	Low	Low	Low	[6]
Carapetis *et al.* 2014	Moderate	Low	Moderate	Low	Low	Low	Moderate	[7]
Linnér *et al.* 2014	Moderate	Low	Moderate	Low	Low	Low	Low	[8]
Adalat *et al.* 2014	Serious	Low	Moderate	Low	Low	Low	Low	[9]

*Data included in the meta-analysis was corrected for confounding only by limiting the analysis to patients treated with clindamycin.

Supplementary Table 5. Risk of bias in the randomized control trial [20]

Study	Bias in selection	Bias in performance	Bias in detection	Bias in attrition	Bias in reporting	Other bias	Ref.
Darenberg *et al.* 2003	Unclear*	Low	Low	Low	Low	Stopped early	[5]

*Neither random sequence nor allocation concealment reported.

Supplementary Table 6. Summary results of included studies

Study	Mortality in full dataset (i.e. irrespective of clindamycin, GAS or STSS)		Mortality in subgroup of interest (i.e. clindamycin-treated STSS)		Ref.
	Cases	Controls	Cases	Controls	
Kaul *et al.* 1999	7/21 (33%)	21/32 (66%)	6/20 (30%)	9/17 (53%)	[6]
Darenberg *et al.* 2003	1/10 (10%)	4/11 (36%)	1/8 (13%)	3/10 (30%)	[5]
Carapetis *et al.* 2014†	1/14 (7%)	19/70 (27%)	1/13 (8%)	6/24 (25%)	[7]
Linnér *et al.* 2014	3/23 (13%)	22/44 (50%)	3/21 (14%)	11/31 (35%)	[8]
Adalat *et al.* 2014	0/8 (0%)	10/41 (24%)	0/8 (0%)	3/13 (23%)	[9]

Supplementary Table 7. Derivation of subgroup of interest from the propensity-matched case-control analysis in Kadri *et al.* [12]

Study	Total IGAS	Proportion STSS	Proportion treated with clindamycin	Proportion treated with clindamycin & IVIG (i.e. cases)	Proportion treated with clindamycin & not IVIG (i.e. controls)	Ref.
Kadri *et al.* 2016	50*	50/50 (100%)†	49/50 (100%)	26/49 (53%)	23/49 (47%)	[12]

*In total 50 of 228 (21.9%) patients with coding for GAS in the entire study were included in the propensity-matched analysis.
†Only 25 of the 49 patients with coding for GAS and clindamycin in the propensity-matched analysis also had coding for toxic shock syndrome. For the purposes of this analysis, however, we presumed all 49 would have met diagnostic criteria for STSS based on the combination of GAS, vasopressor-dependent shock and necrotising infection.

Supplementary References

13. Haywood CT, McGeer A, Low DE. Clinical experience with 20 cases of group A Streptococcus necrotizing fasciitis and myonecrosis: 1995 to 1997. Plast. Reconstr. Surg. **1999**; 103:1567–1573.
14. Huang YC, Chiu CH, Chang LY, Leu HS, Lin TY. Characteristics of group A streptococcal bacteremia with comparison between children and adults. J Microbiol Immunol Infect **2001**; 34:195–200. Available at: https://www.ncbi.nlm.nih.gov/pubmed/?term=11605811.
15. Norrby-Teglund A, Muller MP, McGeer A, et al. Successful management of severe group A streptococcal soft tissue infections using an aggressive medical regimen including intravenous polyspecific immunoglobulin together with a conservative surgical approach. Scand. J. Infect. Dis. **2005**; 37:166–172.
16. Aronoff DM, Mulla ZD. Postpartum invasive group A streptococcal disease in the modern era. Infect Dis Obstet Gynecol **2008**; 2008:796892–6.
17. McViety J, Horan M, Timmis A, Parkins K. Don't forget IGAS: Lessons learnt from review of 2 peak seasons in the North West and North Wales, UK [Abstract 665]. Paediatric Critical Care Medicine **2014**; 15:115.
18. Chen KYH, Cheung M, Burgner DP, Curtis N. Toxic shock syndrome in Australian children. Arch Dis Child **2016**; 101:736–740.
19. Sterne JA, Hernán MA, Reeves BC, et al. ROBINS-I: A tool for assessing risk of bias in non-randomised studies of interventions. BMJ **2016**; 355:i4919.
20. Higgins JPT, Altman DG, Gøtzsche PC, et al. The Cochrane Collaboration's tool for assessing risk of bias in randomised trials. BMJ **2011**; 343:d5928–d5928.
